# Computer aided detection in prostate cancer diagnostics: A promising alternative to biopsy? A retrospective study from 104 lesions with histological ground truth

**DOI:** 10.1371/journal.pone.0185995

**Published:** 2017-10-12

**Authors:** Anika Thon, Ulf Teichgräber, Cornelia Tennstedt-Schenk, Stathis Hadjidemetriou, Sven Winzler, Ansgar Malich, Ismini Papageorgiou

**Affiliations:** 1 Institute of Diagnostic and Interventional Radiology, Department of Experimental Radiology, Jena University Hospital, Friedrich-Schiller University, Jena, Germany; 2 Institute of Radiology, Suedharz Hospital Nordhausen gGmbH, Nordhausen, Germany; 3 Institute for Pathology, Mühlhausen-Pfafferode, Germany; 4 Department of Electrical Engineering and Informatics, Cyprus University of Technology, Limassol, Cyprus; Norges teknisk-naturvitenskapelige universitet, NORWAY

## Abstract

**Background:**

Prostate cancer (PCa) diagnosis by means of multiparametric magnetic resonance imaging (mpMRI) is a current challenge for the development of computer-aided detection (CAD) tools. An innovative CAD-software (Watson Elementary^™^) was proposed to achieve high sensitivity and specificity, as well as to allege a correlate to Gleason grade.

**Aim/Objective:**

To assess the performance of Watson Elementary^™^ in automated PCa diagnosis in our hospital´s database of MRI-guided prostate biopsies.

**Methods:**

The evaluation was retrospective for 104 lesions (47 PCa, 57 benign) from 79, 64.61±6.64 year old patients using 3T T2-weighted imaging, Apparent Diffusion Coefficient (ADC) maps and dynamic contrast enhancement series. Watson Elementary^™^ utilizes signal intensity, diffusion properties and kinetic profile to compute a proportional Gleason grade predictor, termed Malignancy Attention Index (MAI). The analysis focused on (i) the CAD sensitivity and specificity to classify suspect lesions and (ii) the MAI correlation with the histopathological ground truth.

**Results:**

The software revealed a sensitivity of 46.80% for PCa classification. The specificity for PCa was found to be 75.43% with a positive predictive value of 61.11%, a negative predictive value of 63.23% and a false discovery rate of 38.89%. CAD classified PCa and benign lesions with equal probability (*P* 0.06, *χ*^2^ test).

Accordingly, receiver operating characteristic analysis suggests a poor predictive value for MAI with an area under curve of 0.65 (*P* 0.02), which is not superior to the performance of board certified observers. Moreover, MAI revealed no significant correlation with Gleason grade (*P* 0.60, Pearson´s correlation).

**Conclusion:**

The tested CAD software for mpMRI analysis was a weak PCa biomarker in this dataset. Targeted prostate biopsy and histology remains the gold standard for prostate cancer diagnosis.

## Introduction

Prostate cancer (PCa) is the third most common cancer in the total population, representing approximately 11% of all cancer diagnoses [1]. 55–60% of prostate cancer patients are men over 65 years of age. The disease has an excellent survival rate of 94% in the 1^st^ year, 85% in 5 years and 84% in 10 years [2]. The relative survival rate reaches 100% in the first 5 years and 95% in 15 years [1], provided that cancer will be diagnosed at the local (stage I, II) or the regional stage (stage III), as occurs with more than 80% of the cases [1]. A 5-year survival rate at stage IV (distant disease) of 28–30% renders the importance of early stage clinical diagnosis indisputable [1,2].

In spite of being faced with criticism [3–6], the prostate-specific antigen (PSA) assay [7,8] has been, and continues to be the most popular and widely applied PCa screening method in practice for the last 30 years. However, invasive methods such as prostate biopsy still prevail as the gold standard for preoperative evaluation, risk-assessment and decision-making between active surveillance, new evolving tissue-preserving strategies and more radical approaches for aggressive disease such as whole-gland radiation, chemotherapy and radical prostatectomy [7–12]. Due to the low PSA specificity and a large number of false positives, there is an increasing need for a non invasive imaging PCa biomarker [9,10].

A promising list of laboratory non invasive PCa-biomarkers is currently under validation but none of them are a part of the clinical routine [5,9,10]. Prostate magnetic resonance imaging (MRI) is, on the other hand, a promising biomarker already in clinical routine for diagnosis and preoperative evaluation of prostatic lesions in patients with elevated PSA [13–17]. MRI is superior to other diagnostic methods for providing information about the size, localization and spread of the disease. Furthermore, the available multiparametric MRI-sequence battery (mpMRI), including high-resolution T2-weighted imaging (T2w), diffusion-weighted imaging (DWI) with apparent diffusion coefficient (ADC) maps and dynamic contrast-enhanced (DCE) sequences, allows for precise localization and detailed anatomical and functional description of prostatic lesions and their neighboring structures [13,18–21]. The non invasive character and the high negative predictive value of mpMRI combined with minimal invasive MRI-guided biopsy [22], justify them as attractive diagnostic tools that obviate more invasive diagnostic methods such as the multicore (not lesion-targeted or systematic) mapping biopsy [10].

Although the sensitivity of mpMRI is constantly increasing, especially in line with technical advances in diffusion imaging (16), it does not yet qualify for the accurate differential diagnosis of PCa from indolent alterations with similar MRI features, such as benign prostate hypertrophy [14]. Moreover, the interpretation of mpMRI requires highly qualified, board-certified staff, the supply of which is disproportionally low compared to the increased diagnostic demand of the second most common cancer in males. An increasing body of evidence supports the role of automated mpMRI analysis in the form of Computer-Aided Detection (CAD) methods. CAD systems approach MRI modalities quantitatively and allow for information convergence into statistical pipelines that are adjusted to predict malignancy [13].

A recently commercialized, automated analysis tool for the assessment of prostate cancer in mpMRI (Watson Elementary^™^, Watson Medical, Den Ham, The Netherlands) has achieved high sensitivity and specificity in its first evaluation [23]. The Watson Elementary^™^ method is tuned up to predict the malignancy grade with an mpMRI-based Gleason score correlate, termed Malignancy Attention Index (MAI). In this study, Watson Elementary^™^ was retrospectively assessed in our hospital´s database of 104 prostate lesions with histological ground truth after MRI-guided biopsy, and showed a low sensitivity for PCa detection, which was not superior to the observational diagnosis. Our results are compared with previous related studies.

## Materials and methods

### Ethics

All patient data were derived from the prostate database of the Suedharz Hospital Nordhausen. Data were analyzed retrospectively, fully anonymized, in accordance with the ethical standards laid down in the 1964 Declaration of Helsinki and its amendments as well as with the guidelines of the Ethical Committee for clinical studies of the University of Jena. Due to the retrospective character of the study, the ethical committee has waived the mandate for obtaining a legally effective informed consent from the included subjects. Accordingly, therapeutic decisions were not influenced by the outcome of this study.

### Study design

**Fig 1:**
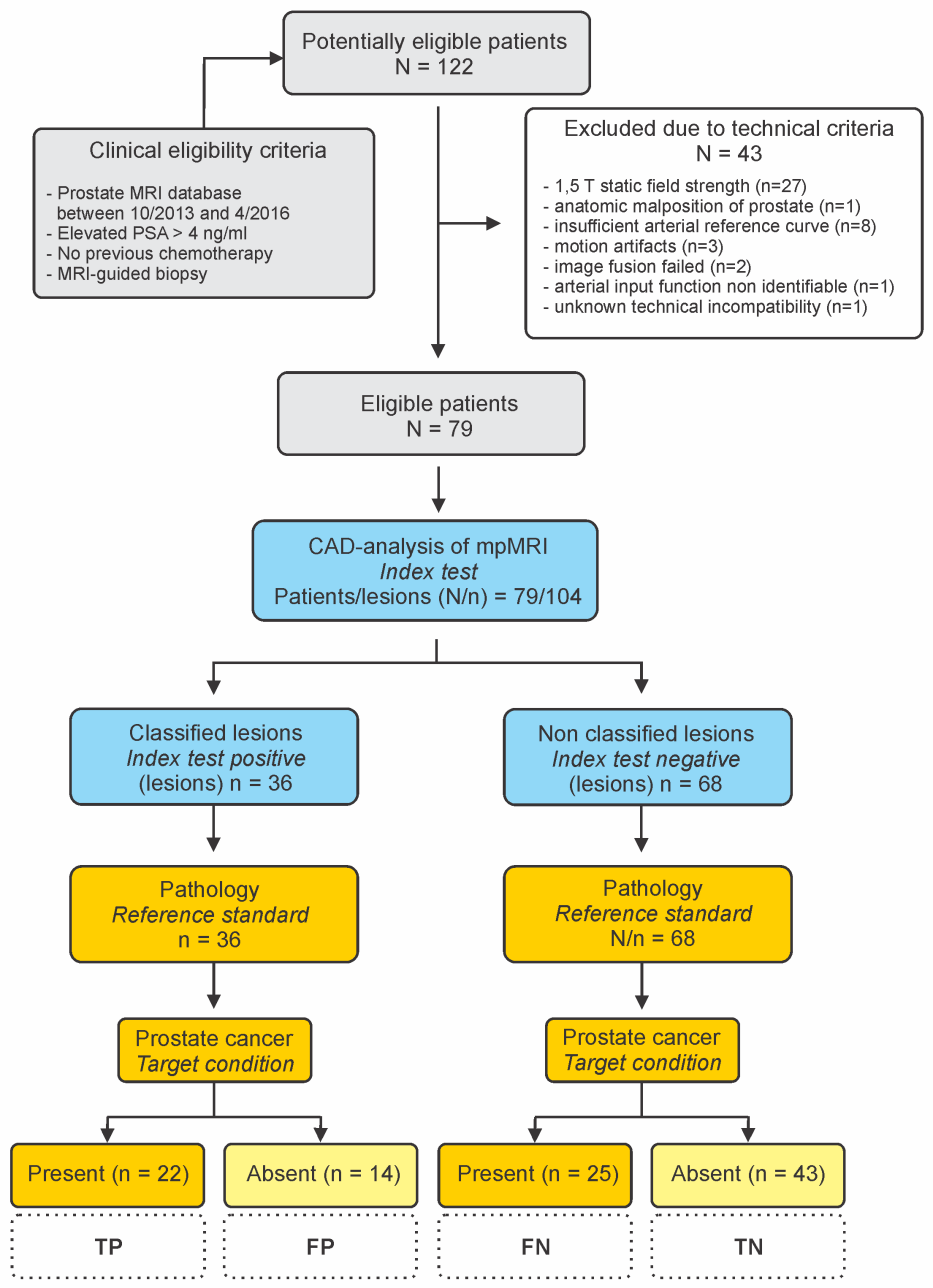
Flow of participants though the study. STARD diagram to demonstrate the flow of participants though the study. Downstream of the “Index test” the analysis is lesion-based due to detection of multiple lesions in some patients. PSA, Prostate Specific Antigen; CAD, Computer-Aided Detection; TP, True Positive; TN; True Negative; FP, False Positive; FN, False Negative.

The evaluation was retrospective for 104 histologically characterized biopsy cores obtained with MRI-guided prostate biopsy (47 malignant, 57 benign) from 79 patients aged 48–80 years (average±σ 64.62±6.64 y.o.), 35 with malignant and 44 with benign lesions, scanned in our department between 10/2013 and 4/2016 (Table 1, Fig 1). All patients were examined with suspicion for prostate cancer based on elevated PSA assay after a negative systematic biopsy and none of them had previously received chemotherapy for prostate cancer treatment. From a total of 122 patients, 43 patients were excluded from this study due to protocol mismatch with the software´s technical requirements: in 27 patients, rejection was due to static field strength inconsistencies (1.5T excluded); in one patient, due to anatomic malposition of the prostate; in eight patients, the arterial reference curve in the DCE sequences was insufficient; three patients were rejected due to motion artifacts; in two cases, the fusion step failed (see later); and in one case the arterial input curve could not be defined. Images from a single patient were not accepted by the software for an unknown reason. No patient- or lesion-related inclusion/exclusion criteria applied.

**Table 1:** Patient demographics and database description.

	Age (y)	*N*	*n*	Classified (%; n)	PIRADS V01 (%; n)			PIRADS V02 (%; n)				Histology
					3	4	5	2	3	4	5	
**Total**	64,61±6,64	79	104	35; 36								
**Malignant**	61,64± 0	1	2	0; 0	0; 0	50; 1	50; 1	0; 0	0; 0	50; 1	50; 1	Gleason 5
	65,70± 6,01	6	7	14; 3	0; 0	43; 3	57; 4	0; 0	0; 0	43; 3	57; 4	Gleason 6
	64,67± 5,12	18	25	55; 12	0; 0	40; 10	60; 15	0; 0	0; 0	64; 16	36; 9	Gleason 7
	65,97± 10,57	5	6	14; 3	0; 0	17; 1	83; 5	0; 0	0; 0	67; 4	33; 2	Gleason 8
	69,06± 5,32	4	5	18; 4	0; 0	0; 0	100; 5	0; 0	0; 0	0; 0	100; 5	Gleason 9
	62,54±0	1	2	0; 0	0; 0	50; 1	50; 1	0; 0	0; 0	100; 2	0; 0	Gleason 10
**Total malignant**	65,38±6,18	35	47	61; 22	0; 0	34; 16	66; 31	0; 0	0; 0	55; 26	45; 21	
**Benign**	63,79± 4,77	10	15	21; 3	0; 0	73; 11	27; 4	7; 1	7; 1	60;9	27; 4	Prostatitis
	62,78± 8,75	15	20	36; 5	15; 3	55; 11	30; 6	0; 0	10; 2	80; 16	10; 2	BPH
	67,52± 6,23	4	6	14; 2	0; 0	50; 3	50; 3	0; 0	0; 0	83; 5	17; 1	Prostatic tissue
	65,99± 8,90	2	2	7; 1	0; 0	100;2	0; 0	0; 0	50; 1	50; 1	0; 0	Atrophy
	63,61± 0	1	1	0; 0	0; 0	100;1	0; 0	0; 0	0; 0	100; 1	0; 0	FMD
	64,25± 7,40	12	13	21; 3	0; 0	54; 7	46; 6	8; 1	8; 1	70; 9	15; 2	ASAP
**Total benign**	64,01±6,73	44	57	39; 14	5; 3	61; 35	33; 19	4; 2	9; 5	72; 41	16; 9	

Software evaluation was based on a series of 104 histologically confirmed prostatic lesions from 79 patients. Multiparametric MRI evaluation was performed in diagnostic series acquired within 3 months before the biopsy. Lesions have been scored according to the Prostate Imaging Reporting And Data System (PI-RADS^™^) v1 and -v2. All values are reported in mean, σ. N, patients; n, lesions; BPH, Benign Prostate Hyperplasia; FMD, Fibromuscular Dysplasia; ASAP, Atypical Small Acinar Proliferation.

All lesions were graded by 2 radiologists; one with intermediate experience, and one board-certified radiologist, according to the Prostate Imaging Reporting And Data System (PI-RADS^™^) v1 and -v2. MRI-guided transrectal needle biopsies were always performed in less than 3 months post diagnosis. Histological characterization and Gleason grading on H&E stained sections followed.

Watson Elementary^™^ (installed on 11.5.2016 by Watson Medical, Den Ham, The Netherlands) was tested with a manual generation of regions of interest (ROI) in diagnostic MRI series encompassing targeted biopsy cores. The diagnostic accuracy hypothesis tested the ability of CAD to detect known, manually drawn lesions. Therefore all lesions without histological ground truth, including new lesions identified by the software, were neglected and did not influence the statistics of the current study.

Evaluation of the diagnostic accuracy followed with estimation of the sensitivity, specificity, Positive Predictive Value (PPV) and False Negative Rate (FNR). Implementation of Receiver Operatic Characteristic (ROC) analysis and estimation of the Area Under the Curve (AUC) allowed for the definition of optimal cut-off diagnostic values using Youden statistics (see also *Statistics*).

### MRI-guided prostatic biopsy

Malignancy suspect lesions according to PI-RADS^™^ scoring were transrectally biopsied under stereotactic MRI-guidance with a Philips Ingenia 3.0T MR system using a dStream Torso body coil with Flex Coverage anterior and posterior coils that allow for a 32-channel, 60 cm body coverage (Philips North America Corporation, Andover, MA USA).

Briefly, a stereotactic needle-frame with x-y-z freedom was fixed onto the patient table. Target lesions were (re-)allocated and the sampling position was planned with the frame-dedicated software (DynaCAD, Invivo Corporation, Gainesville, FL, USA). The accurate position of the biopsy needle-tip was confirmed with a T2w TSE HR sequence.

### Image acquisition for mpMRI

**Fig 2:**
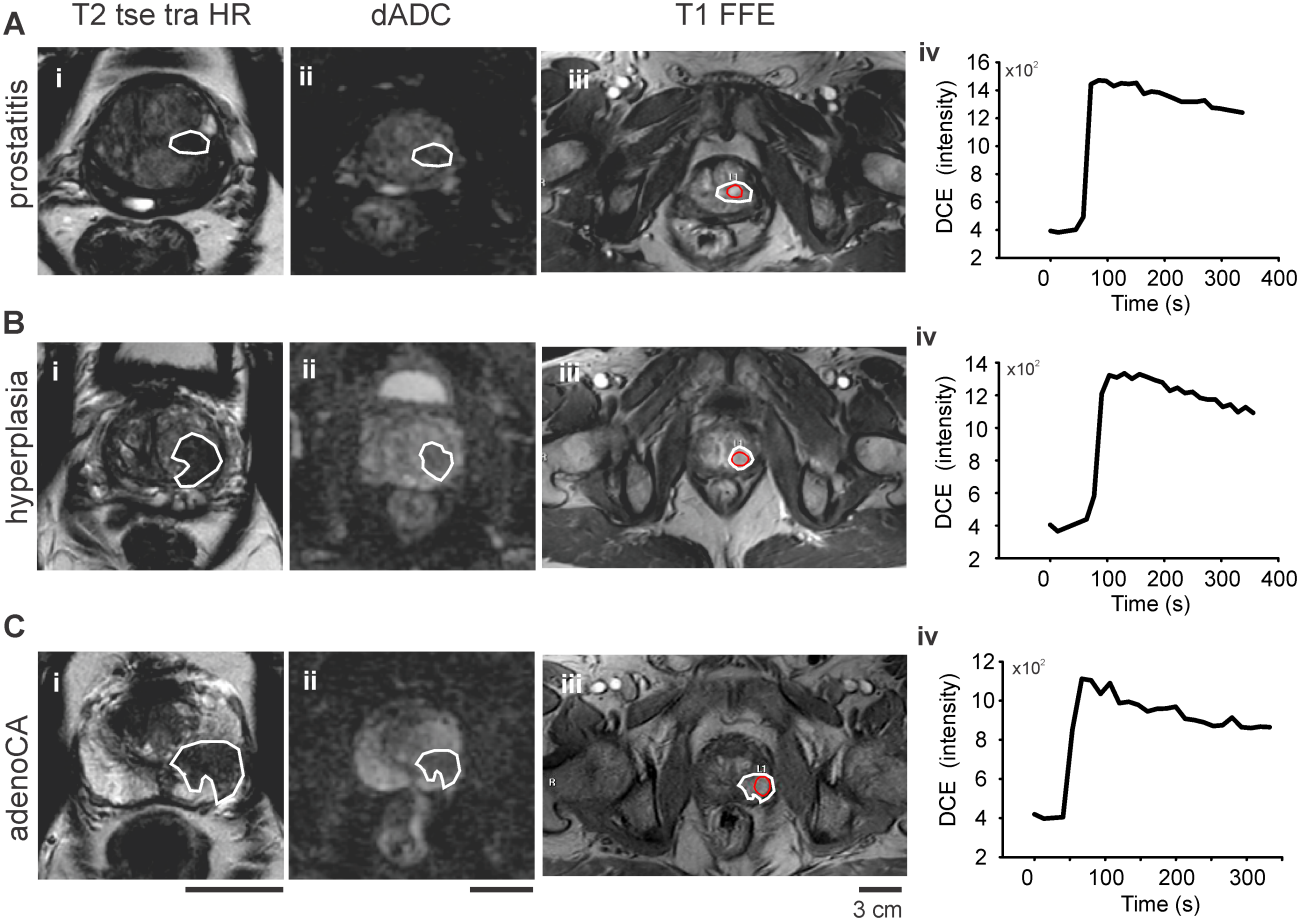
Sequences used for multiparametric MRI (mpMRI) imaging according to PI-RADS^™^. Sample images from lesions (white ROIs in A-C) histologically characterized as (A) prostatitis (B) hyperplasia (BPH) and (C) adenocarcinoma (PCa). (i) T2w TSE images, (ii) apparent diffusion coefficient (ADC) tables based on DWI b0-100-500-800-1000 s/mm^2^ (iii) T1w FFE with enhancement and (iv) sample Dynamic Contrast Enhanced (DCE) curves of the lesion core, defined as red colored ROI in the T1w FFE images. Scale bar 3cm. For MRI sequence parameter details see Table 2.

Both biopsies and diagnostic imaging were performed on the same Philips Ingenia 3.0T MR-system within a time interval of 3 months. The following axially orientated image set was used for CAD analysis (Table 2):

1. T2 weighted high-resolution Turbo Spin Echo imaging (T2 TSE HR) (Fig 2Ai, 2Bi and 2Ci)
2. Diffusion weighted Echo Planar Imaging (DWI EPI) at 5 different b-values (b0-100-500-800-1000)
3. T1 weighted Fast Field Echo (T1-FFE) with dynamic contrast enhancement (DCE) in 25 repetitions with 13.35 s temporal resolution and 7 s delay, which corresponded to at least two baseline (zero contrast) points. A body weight-adjusted bolus of gadoteridol 0.1 mmol/kg (ProHance^®^, Bracco S.p.A., Milan, Italy) was injected at 3 ml/s flow rate. Watson Elementary^™^ provides only visual access to the DCE curve in.pdf format, which is not indicated for publication purposes. Therefore, for demonstration purposes, example DCE curves from selected lesions were manually exported (Fig 2Aiii and 2Aiv, 2Biii and 2Biv, 2Ciii and 2Civ).

**Table 2:** Sequences used for multiparametric magnetic resonance imaging.

Parameter/Sequence	T2 TSE HR	DWI EPI 20	T1 FFE with DCE
TR(ms)/TE(ms)	4811/120	4227/63	5.5/1.93
Flip angle (°)	90	90	15
EPI factor		single shot technique	
Number of averages	1	4	1
b values s/mm^2^		0-100-500-800-1000	
Slice thickness (mm)	3	3	3
FOV AP×FH×RL (mm)	260×75×160	256×75×256	262×120×262
Voxel size AP×RL×FH (mm^3^)	0.65×0.65×3	1.8×1.8×3	1.02×1.41×3
Aquisition time (min:s:ms)	3:12	5:25	0:13:35 pro sequence, 25 dynamic sequences 5:31 in total
Enhancer	Gadoteridol (ProHance^®^) 0.1 mmol (0.2 ml)/kg, injection rate 3 ml/s		

Imaging was performed at 3T field strength. TR, repetition time; TE, echo time; EPI, Echo-Planar Imaging factor and number of k-spaces collected in a single shot; FOV, Field Of View; AP, Anterior to Posterior; FH, Foot to Head; RL, Right to Left.

### Description of Watson Elementary^™^

Watson Elementary^™^ software implements a fully automated 3-step-method, previously reported in detail [23]. Below is a brief description of its image processing steps:

1. Affine image co-registration
2. Pixel detection step for feature extraction and non linear processing, such as ADC maps from DWI sequences (Fig 2Aii, 2Bii and 2Cii), kinetic parameters of the DCE-profile such as K_*trans*_, V_*e*_ and K_*ep*_ according to Tofts´ pharmacokinetic model [24,25] and normalization of T2w images based on a rectangular prostatic reference volume for calculation of first and second order texture features.
3. Feature classifier with 3 logical steps: Step 1 defines the dimension of predictor space and the transformation parameters. Step 2 is a linear summation parameter that integrates step 1 information to construct a scalar map. Step 3 features an error-feedback method, which has been trained by a supervised learning process to achieve a congruence of the scalar value (step 2) with the Gleason grade.

The final output of the feature classifier is a pixel-based malignancy prediction score, ranging from 0 to 1, termed as Malignancy Attention Index (MAI). Watson Elementary^™^ constructs a malignancy prediction heatmap with high MAI values represented in warm colors. This map is projected onto T2w images, thus anatomically highlighting suspect lesions. Moreover, MAI pixel values of each manually defined lesion are automatically sorted in a histogram (malignancy prediction histogram). Histogram shape, mean and median MAI have been suggested as PCa biomarkers [23].

### Lesion definition and evaluation of the volume of interest

**Fig 3:**
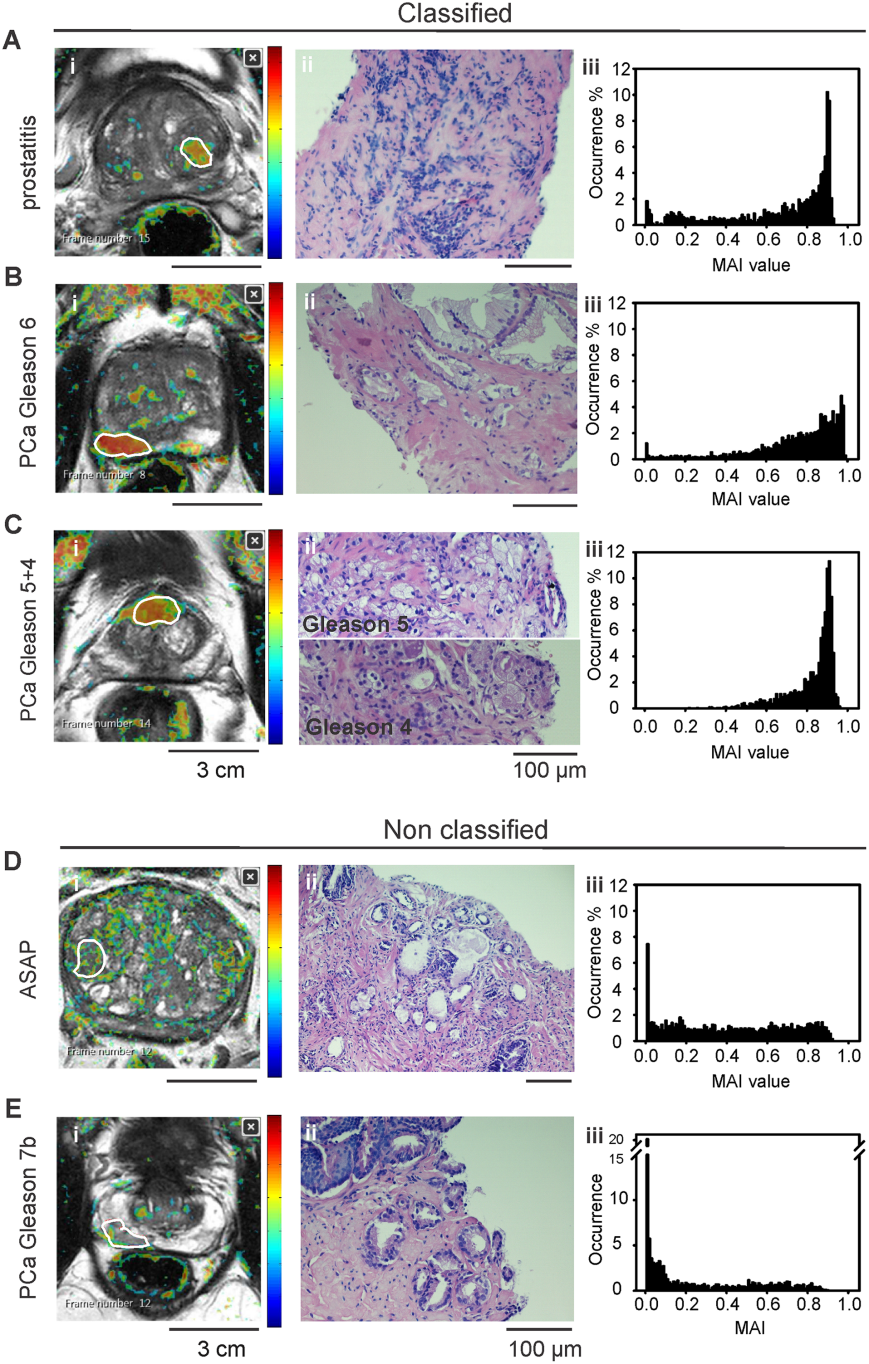
Automated mpMRI classification and histological ground truth. Target lesions were manually drawn by human observers. Sample images for (A-C) classified and (D, E) non classified PCa and benign biopsy cores. (A) Prostatitis, (B) adenocarcinoma (PCa) Gleason grade 6, (C) PCa Gleason grade 9, (D) atypical small acinar proliferation (ASAP) and (E) PCa Gleason grade 7. From left to right: (i) T2w with MAI-heatmap and outlined lesions (white line), (ii) Hematoxylin-Eosin histopathology of the corresponding biopsy cores and (iii) MAI histograms. Warm colors in MAI heatmaps (i) represent higher values in a scale 0–1. Classified lesions revealed a “warm-colored” MAI-map and a left-skewed histogram.

Target lesions, i.e. lesions that have been selected for MRI-guided biopsy based on their PI-RADS score, were evaluated retrospectively, after PI-RADS scoring and after the histological report of the biopsy. Lesions were manually defined in T2w images (for the transitional zone) and ADC maps (for the peripheral prostate zone) according to the biopsy needle position by a third radiologist (IEP) blinded to the PI-RADS scoring and the histological identity to avoid bias in sampling. Data analysis was performed by a medical student (AT) and a radiologist (IEP), both blinded to the PI-RADS scoring and histological identity to avoid bias in lesion classification. Watson Elementary^™^ allows for definition of consecutive ROIs in 2D planes to form a 3D lesion volume of interest. The system generates a probabilistic heatmap (with the MAI values) for each section, which is projected onto the corresponding T2w image. The predefined ROIs were copied from the imaging series to the probabilistic map and the system generated for each predefined lesion (sum of ROIs) a feature summary in a.pdf including (i) a histogram of MAI values, (ii) the average DCE curve (iii) the lesion volume [26]. User access to the ground data is possible only for the probabilistic histograms for each region and not for the intermediate meta-data such as DCE curves, which are only graphically displayed in.pdf format. The classification of lesions was performed visually, by evaluating (i) whether the heatmap of the lesion is distinguishable from the background and (ii) the skewness of the MAI histogram (Fig 3). Classification was performed by a radiologist (IEP) and a medical student (AT), both blinded to the PI-RADS scoring and histological identity of the lesions. In the first classification step observers made no assumption about the histological identity. After classification, the histological identity was unmasked to calculate the specificity and the sensitivity of the software for malignant lesions.

### Statistics

The Statistical Package for the Social Sciences (SPSS Version 21, IBM, Armonk, NY, USA) was used for statistical analysis and graphical plotting. Data were screened for normality using the Shapiro-Wilk test. Values are expressed as median/IQR (interquartile range) and rounded up to the second decimal place unless otherwise stated. Statistical significance was tested using the t-test or the ranksum Mann-Whitney test for unpaired data. MAI score between groups were compared with Kruskal-Wallis ANOVA on ranks with Dunn´s posthoc test. Linear correlations were tested with the Pearson product moment correlation coefficient. Continuous probability distribution, as well as independence of nominal data, was tested by means of the chi-squared test. ROC curves were calculated for MAI median, MAI mean and MAI median-to-mean ratio as skewness index as well as for ADC values and PI-RADS reading scores. Statistical significance was set at *P* <0.05.

## Results

### Automated feature extraction: Detection of the arterial input function

The first step in image processing by Watson Elementary^™^ is feature extraction. For the DCE kinetic analysis, the tissue signal is normalized on the perfusion curve of the common femoral artery (arterial input function). Arterial detection is semi-automated and has to be manually confirmed. The correct artery position was automatically defined in 61 (77.22%) patients; in the remaining 18 (22.78%), it had to be manually reassessed.

### CAD-sensitivity and specificity

All 104 biopsy cores from 79 patients were scored according to PI-RADS^™^ in the initial diagnostic dataset (Table 1). Datasets acquired prior to the establishment of PI-RADS^™^ v2 were re-scored post hoc so that PI-RADS^™^ v1 and -v2 scores were available for all included cores. Switching between PI-RADS versions, as a consequence, over- or underscored some lesions. Although PI-RADS 2 lesions were not subjected to a biopsy, some were included in the analyzed database. Those lesions were biopsied as PI-RADS 3 or 4 according to PI-RADS^™^ v1 and then underscored to PI-RADS 2 after the introduction of PI-RADS^™^ v2 (Table 1).

In the joined MAI/T2w images (Fig 3A–3E, left panels), we evaluated whether the heatmap of our target lesions was visually distinguishable from the background, i.e. whether the lesion could be detected in the MAI/T2w heatmap without the use of ADC maps. Warm-colored ROIs with a characteristic left-skewed MAI histogram were evaluated as classified (detected) lesions (Fig 3A–3C), in sharp contrast to the flat or right-skewed MAI histogram shape of the non classified (cold-colored) ROIs (Fig 3D and 3E). In previous work, a MAI-max cut-off value of 0.6 was used as criterion for malignant lesion classification, based on the assumption that MAI linearly correlates with the Gleason score [23]; this method was not applicable in our study because almost all lesions, regardless of their histogram shape and histological identity, showed a maximum MAI value higher than 0.6 (Supporting information, S1).

**Fig 4:**
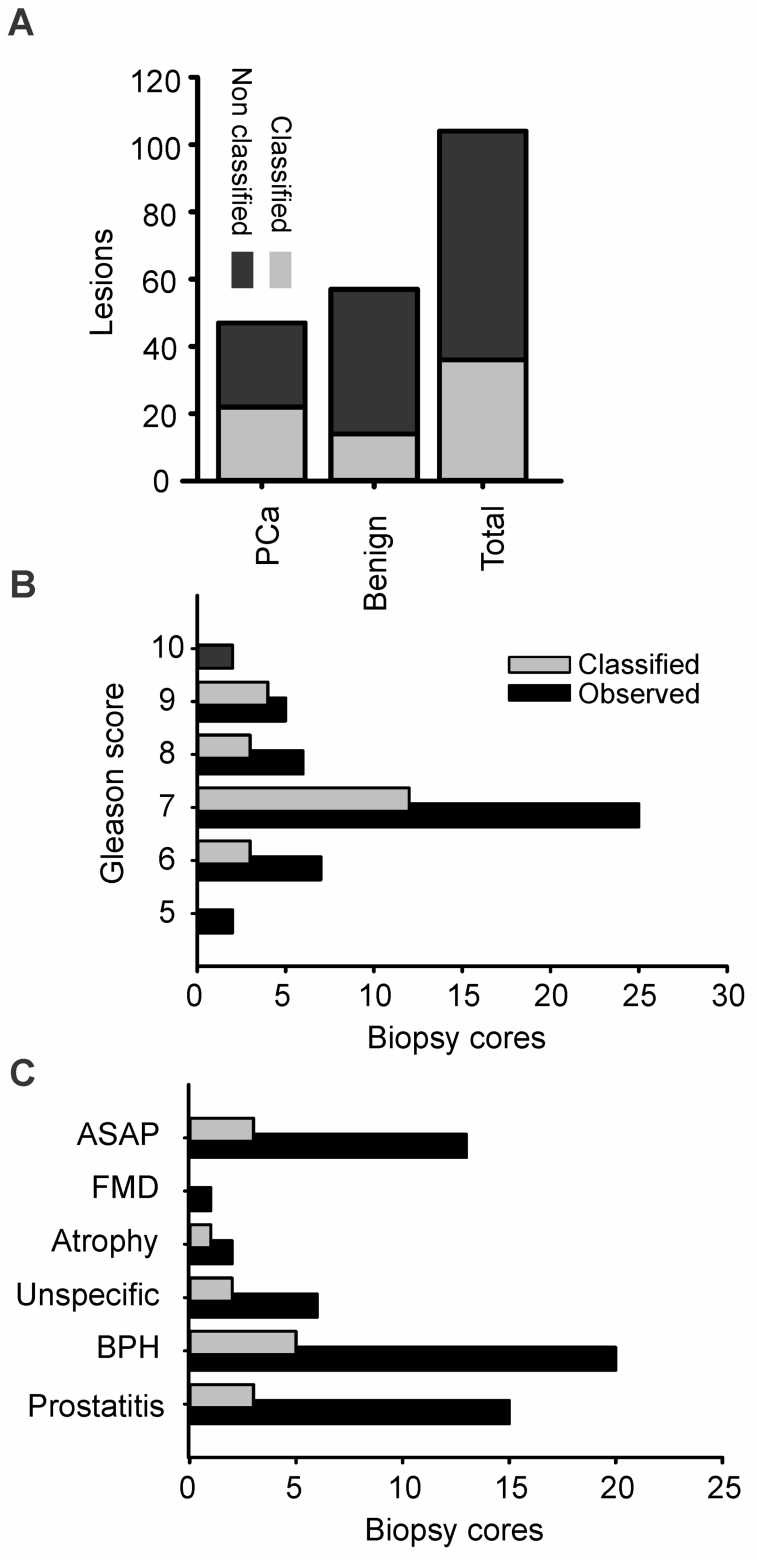
Classification of malignant and benign biopsy cores. (A) Proportion of classified to non classified malignant and benign lesions. From 104 mixed type cores 36 were classified, from those (classified/total) 14/57 were benign and 22/47 malignant. (B) The classification was independent of the Gleason grade, P 0.713 R 0.193 Pearson correlation (C) Classification of benign lesions. ASAP, Atypical Small Acinar Proliferation; FMD, Fibromuscular Dysplasia; BPH, Benign Prostatic Hyperplasia.

From 47 histologically confirmed malignant (PCa) and 57 benign lesions, 22 PCa and 14 benign lesions were classified (Fig 4A and Table 3). The deduced CAD-sensitivity for prostate malignancy in our series was 46.81% with a specificity of 75.44% and a PPV of 61.11% (Table 4).

**Table 3:** Identity of classified and non classified lesions.

Patient *N* = 79	Classified	Non-classified	Total
**Malignant (PCa)**	22	25	47
**Benign**	14	43	57
**Total**	36	68	104
	*P* = 0.06, Chi square test		

The classified malignant cores are the true positives (TP), the non classified malignant cores are sorted as false negatives (FN), the classified benign cores as false positives (FP) and the non classified benign cores as true negatives (TN). Chi square test returns equal occurrence of the outcomes for the two groups, P 0.06, χ^2^ test.

**Table 4:** CAD sensitivity and specificity for PCa detection.

False negative rate (FNR)	0.53 lesions/patient
Sensitivity	46.81%
Specificity	75.44%
Positive predictive value (PPV)	61.11%
Negative predictive value (NPV)	63.24%
False positive rate (FPR)	24.56%
False detection rate (FDR)	38.89%

The hypothesis whether CAD preferentially classifies malignant over benign cores was tested with a chi-squared test, which revealed independent classification of benign and malignant biopsy cores, albeit with a slight trend towards the classification of malignancy, *P* 0.06 (Table 3). Furthermore, we questioned whether CAD sensitivity leaned towards a particular histological identity, a particular Gleason malignancy grade or a specific benign condition. All malignant lesions (*n* 47) were typified as acinar adenocarcinoma of various Gleason grades from 5 to 10. As graphically demonstrated in (Fig 4B), CAD-sensitivity was not influenced by the malignancy grade, *P* 0.713 *R* 0.193 Pearson correlation. Among false positives (i.e. benign cores falsely classified) (Fig 4C), 5/14 lesions (35.71%) corresponded to benign prostate hyperplasia, followed by prostatitis and atypical small acinar proliferation with 3/14 lesions, 21.43% each.

### Malignancy attention index as biomarker

In a previous study [23], MAI was proposed as a potential PCa biomarker and MAI heatmaps/histograms as potential core malignancy profiles (Fig 3).

**Fig 5:**
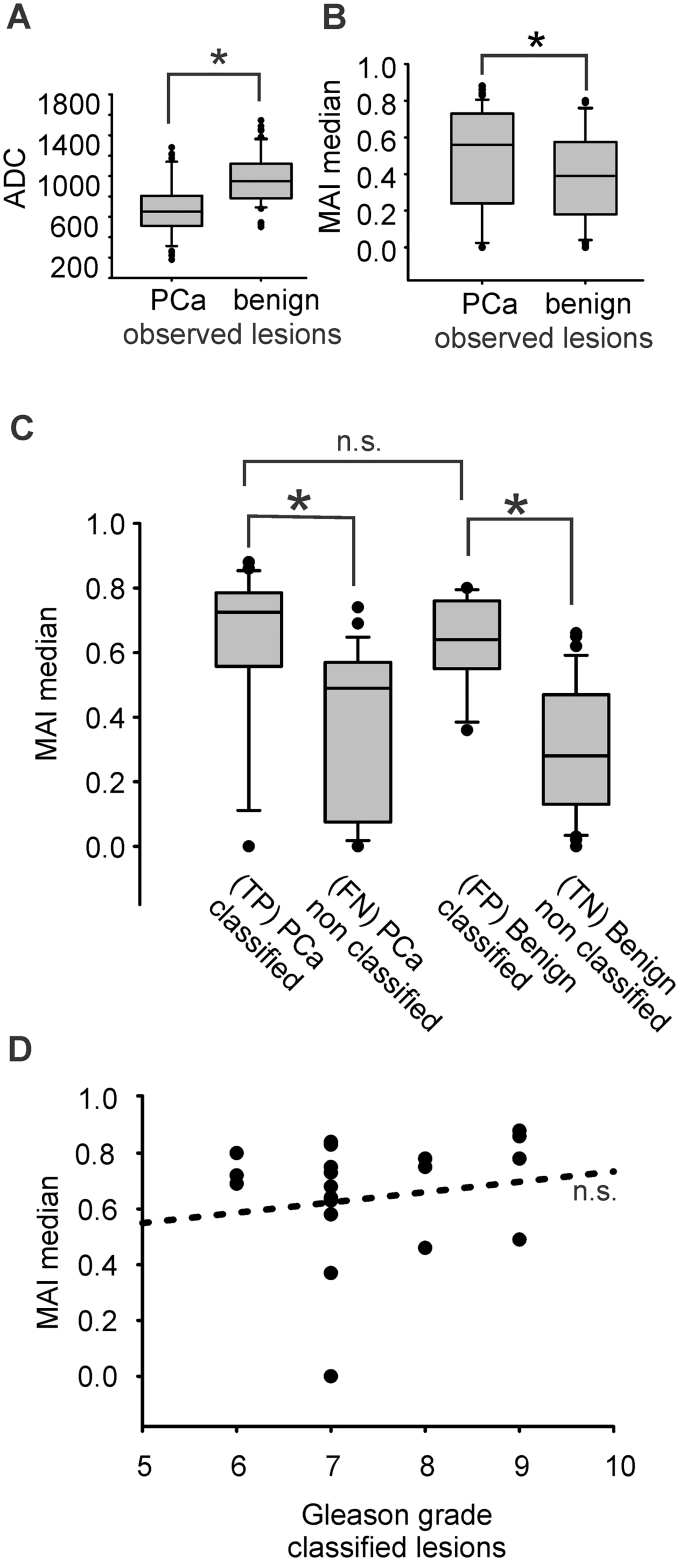
MAI as malignancy biomarker and MAI correlation with the Gleason grade. (A) Average ADC values (x10^-6^ mm^2^/s) of observed lesions, 650/295 (median/IQR) for malignant and 950/339.5 for benign, *N* (malignant/benign) 47/57, P < 0.0001 Mann-Whitney U-test (B) MAI of observed lesions, 0.56/0.23 (median/IQR) for malignant and 0.39/0.18 for benign lesions, *N* (malignant/benign) 47/57, *P* 0.023 Mann-Whitney U-test. (C) MAI in benign, malignant, classified and non classified biopsy cores, *P* < 0.001 Kruskal-Wallis ANOVA on ranks, * *P* < 0.05 Dunn´s post hoc test (D) No significant correlation between MAI and Gleason grade, *P* 0.522, *N* 22, *R* 0.144, Pearson´s product moment correlation; n.s., non significant.

In the database used in our study, malignant lesions showed the expected ADC dip compared to benign ones, with an ADC (median/IQR) of 650/295 (x10^-6^) mm^2^/s for malignant and 950/340 (x10^-6^) mm^2^/s for benign foci, *P* < 0.001 Mann-Whitney U-test (Fig 5A). This is important because ADC values are widely accepted as the most relevant malignancy feature, especially for the peripheral zone, and are thus highly ranked by CAD-classifiers [27–32]. Median MAI values were selected as the most representative descriptive parameter of skewed histograms. The comparison between all observed (classified and non classified) benign and malignant biopsy cores indeed revealed a significantly lower MAI score for benign lesions, with (median/IQR) 0.39/0.18 compared to malignant ones 0.56/0.24, *P* 0.023 Mann-Whitney U-test (Fig 5B).

MAI score was, as expected, significantly higher in classified compared to non classified lesions regardless of identity, *P* < 0.05 Kruskal-Wallis ANOVA on ranks with Dunn´s post hoc test. However, classified PCa and benign cores did not show any significant MAI difference, *P* < 0.05 Kruskal Wallis ANOVA on ranks with Dunn´s post hoc test (Fig 5C).

Furthermore, we tested whether MAI score qualifies for a Gleason´s grade predictor using the Pearson´s test. Within the 22 classified PCa biopsy cores, MAI did not show any significant correlation with Gleason grade, *P* 0.52 *R* 0.14 Pearson product moment correlation (Fig 5D).

**Fig 6:**
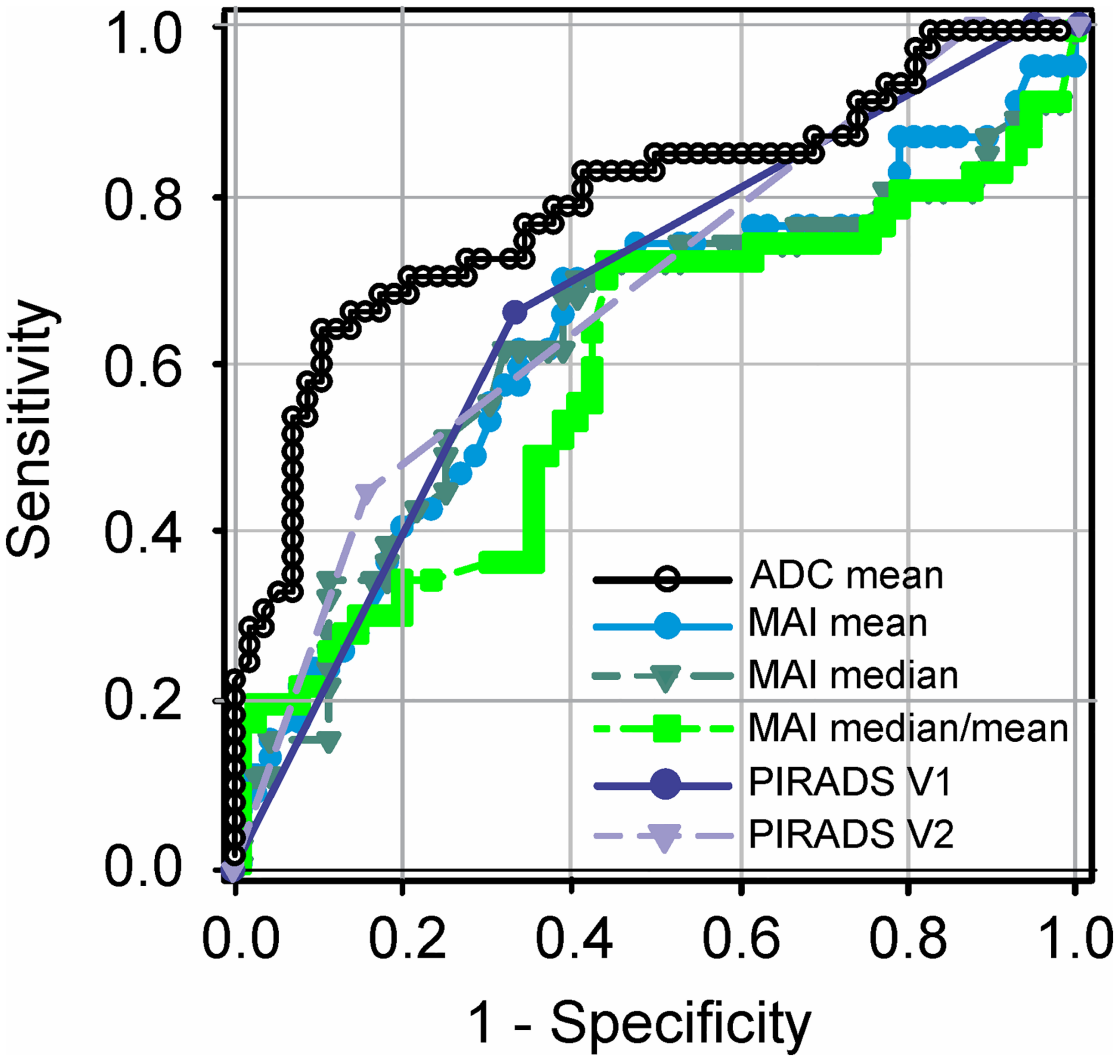
Receiver operating characteristic (ROC) trade-off curve for ADC, MAI and PI-RADS. ADC mean (black), MAI median (dark green), MAI mean (light blue) and MAI median/mean ratio (green). MAI is compared with the ADC performance alone and with the observer´s performance according to PI-RADS^™^ v1 (dark blue) and PI-RADS^™^ v2 (violet). The area under the curve (AUC) is 0.64±0.057 (mean, SEM) with 95% CI 0.53–0.75 and *P* 0.02 for MAI mean, 0.63±0.058 with 95% CI 0.52–0.74 and *P* 0.02 for MAI median and 0.59±0.058 with 95% CI 0.47–0.70 and *P* 0.13 for the MAI median/mean ratio. Corresponding values for the mean ADC lesion value are AUC 0.79±0.05 with 95% CI 0.70–0.88, *P* < 0.0001. Observer´s performance for PI-RADS^™^ v1 was AUC 0.67±0.05 with CI 0.58–0.76, *P* 0.003 and for PI-RADS^™^ v2 AUC 0.68±0.04 with CI 0.59–0.76, P 0.002. *N* malignant/benign cores 47/57. MAI and PI-RADS (v1, v2) reveal comparable performances in malignancy detection, *P* 0.60 for MAI vs PI-RADS v1 and P 0.53 for MAI vs PI-RADS v2, chi-squared test. ADC is superior to MAI in malignancy prediction, *P* 0.04, chi-squared test.

ROC analysis of median and mean MAI as malignancy predictors revealed rather poor results with an area under curve ± standard error of the mean (AUC±SEM) 0.63±0.06 (95% CI 0.52–0.74), *P* 0.02 for MAI median and 0.64±0.06 (95% CI 0.53–0.75), *P* 0.02 for MAI mean. The predictive outcome of the median/mean ratio as skewness index was not significant with AUC 0.59±0.06 (95% CI 0.47–0.70), *P* 0.12 (Fig 6, Table 5). MAI did not significantly improve the board certified reader´s accuracy using the American College of Radiology (ACR) and European Society for Uroradiology (ESUR) standards PI-RADS^™^ v1 (AUC 0.67±0.05 with 95% CI 0.58–0.76, *P* 0.003) and PI-RADS^™^ v2 (AUC 0.68±0.04 with 95% CI 0.59–0.76, *P* 0.002). The optimal MAI median cut-off point estimated with Youden statistics was 0.54 with a sensitivity of 61.7% (95% CI 46.38–75.49%) and specificity of 68.42% (95% CI 54.76–80.09%) (Fig 6 and Table 5). By setting an optimized cut-off point for MAI mean, however, we could improve the sensitivity and specificity up to 70.21% / 61.4% (95% CI 55.11–82.66% and 47.57–74%, respectively) (Fig 6 and Table 5). Moreover, analysis of the ADC value alone showed a stronger predictive behavior compared to the software-calculated MAI with AUC 0.79±0.05 (95% CI 0.70–0.88) and *P* 0.04 compared to MAI, chi-squared test (Fig 6).

**Table 5:** Receiver operating characteristic (ROC) analysis for MAI and PI-RADS.

	Area under the curve (AUC)				*P*	Cut-off	Sensitivity at cut-off			Specificity at cut-off		
	AUC	SEM	95% CI				Sensitivity	95% CI		Specificity	95% CI	
**MAI mean**	0.64	0.06	0.53	0.75	0.02	0.435	70.21	55.11	82.66	61.4	47.57	74
**MAI median**	0.63	0.06	0.47	0.7	0.02	0.535	61.7	46.38	75.49	68.42	54.76	80.09
**PIRADS v1**	0.67	0.05	0.58	0.76	0.003							
**PIRADS v2**	0.68	0.04	0.59	0.76	0.002							

Sensitivity and specificity for MAI and PI-RADS as estimated with Receiver Operating Characteristic (ROC) curve analysis and Youden statistics. MAI mean and MAI median correspond to the mean and median values of the MAI histogram describing each lesion. PI-RADS v1 and -v2 are evaluated as malignancy prediction indices compared to MAI. *N* malignant/benign cores 47/57. AUC, Area Under Curve; SEM, Standard Error of Mean; CI, Confidence Interval; PI-RADS, Prostate Imaging Reporting And Diagnosis System.

**Fig 7:**
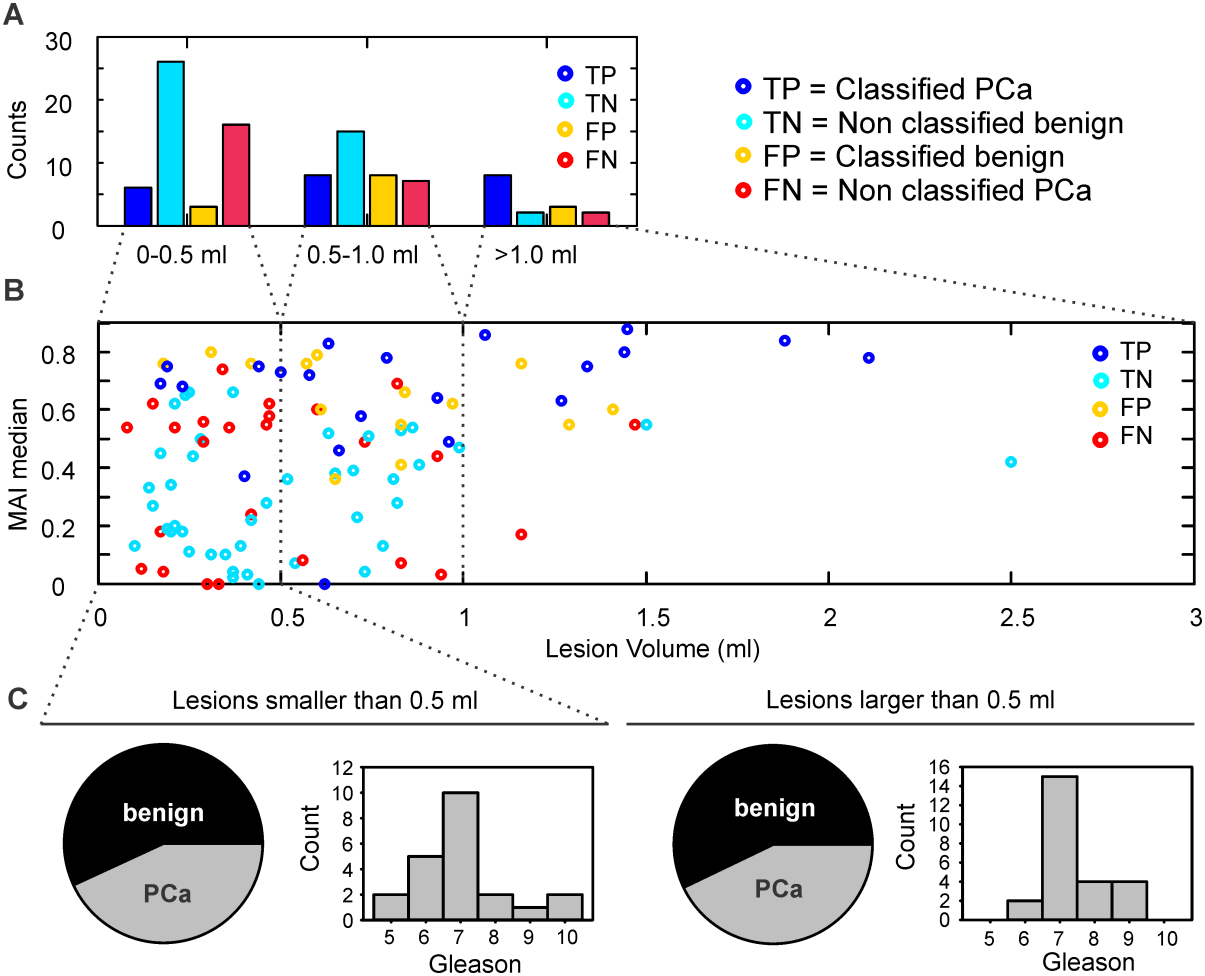
CAD performance is reliable for lesions larger than 1 ml. (A) Counts of classified and non classified cores in relation to their volume. Volume distribution of all lesions (number, %): 0–0.5ml (51, 49.04), 0.5–1.0ml (38, 36.54) and larger than 1.0ml (15, 14.42). True positive (TP, blue) lesions include classified PCa, true negatives (TN, cyan) are the non classified benign cores, false positives (FP, yellow) are the classified benign cores and false negatives (FN, red) the non classified PCa. The CAD-sensitivity increases and the number of FN decreases towards larger lesion volumes: (sensitivity % / FNR %) 27.27/31.37 for 0–0.5ml lesions, 53.33/18.42 for 0.5–1.0ml lesions and 80.00/13.33% for lesions larger than 1.0ml. (B) MAI score with lesion volume. A strong trend for a positive correlation between lesion size and MAI-score was found for TP lesions (P 0.057, Pearson´s correlation) but not for any of the remaining categories (TN, FP and FN, P > 0.1, Pearson´s correlation). (C) Lesions smaller than 0.5 ml show the same malignancy incidence and comparable aggressiveness compared to larger lesions (Ci) Lesions smaller than 0.5 ml (number, %) benign (29, 56.86) malignant (22, 43.14), (Cii) Gleason histogram for malignant lesions smaller than 0.5 ml, (Ciii) Lesions larger than 0.5 ml (number, %) benign (28, 57.14) malignant (21, 42.86), (Civ) Gleason histogram for malignant lesions larger than 0.5 ml.

Guided by the hypothesis that Watson Elementary^™^ might be more specific for particular lesion locations and sizes, we tested for possible predilection towards the peripheral or the central zone of the prostate gland. In the transitional prostate zone CAD has classified 10 out of 25 histologically confirmed PCa; whereas in the peripheral zone, 12 out of 22, hence with no apparent influence on the performance (*P* 0.481 chi-squared test). The lesion volume, however, had a significant influence on the CAD-performance. Amongst lesions smaller than 0.5ml (Fig 7A) the vast majority was not classified (sensitivity 27.27% and FNR 31.37%). For intermediate size cores of 0.5ml-1.0ml, the CAD revealed an improved performance (sensitivity 53.33% and FNR 18.42%) and false negatives were minimized for lesions larger than 1.0 ml (sensitivity 80%, FNR 13.33%). As expected, lesion volume was independent of Gleason grade, *R* 0.18 *P* 0.15 Pearson´s correlation. It´s worth noticing that it is more crucial to eliminate the number of FN than the number of FP because the therapeutic consequence for the patient would be an undiagnosed PCa in the first case, compared to an unnecessary biopsy in the second case. In this context, CAD-performance is satisfactory for lesions larger than 1.0ml (Fig 7A). In Fig 7B, the MAI score of classified and non classified cores is plotted with the lesion volume. There is a strong trend for a positive correlation between lesion size and MAI-score for TP lesions (*P* 0.057 Pearson´s correlation) but not for any other category (TN, FP and FN, *P* > 0.1 Pearson´s correlation). We questioned the clinical significance of lesions smaller than 0.5 ml, which make up 49.04% of our database. Interestingly, the malignancy incidence between lesions smaller than 0.5 ml, and those that were larger, was identical (Fig 7Ci and 7Ciii) with approximately 43% probability of malignancy in both groups. Moreover, within malignant lesions, we observed comparable PCa aggressiveness in terms of Gleason grade (Fig 7Cii and 7Civ), with high-grade cancers being equally possible in both small and larger lesions.

In summary, MAI is a weak PCa biomarker, especially for lesions smaller than 0.5ml in either the transitional and peripheral zone, regardless of lesion aggressiveness.

## Discussion

This study aims to emphasize the growing necessity for commercialized prostate mpMRI CAD-software tools for the radiological, and perhaps urological, praxis. By retrospectively testing 104 lesions (47 malignant, 57 benign) in a series of 79 patients, a commercialized prostate CAD, Watson Elementary^™^, revealed a sensitivity of 46.81% for prostate malignancy, with a specificity of 75.44% and a PPV of 61.11%. Our results considerably differ from previous reports on the same software. Roethke et al. [23] have tested Watson Elementary^™^ in a cohort of 45 patients with 1102 MR/TRUS acquired biopsy cores (76 malignant/1026 benign) and achieved a sensitivity of 85.71% and specificity of 87.50% when setting an optimal MAI mean cut-off threshold for malignancy detection. By setting an optimized cut-off value of MAI mean in our study, we could improve the sensitivity and specificity up to 70.21%/61.4%, which is inferior to the previously reported values but comparable in terms of methodology [23]. This considerably differs from previous promising studies that have established custom-made software tools for mpMRI analysis with high stand-alone accuracy for malignancy detection [33–36]. The group of Litjens et al. [26] achieved a stand-alone accuracy of AUC = 0.89 in a remarkably large database of 347 patients.

The outcome discrepancy between our series and previous testing of the same software [23] could be attested to a variety of causes. In terms of methodology, a previous study applied a combination of systematic and MRI-guided transperineal biopsies, ending up with more probes per patient (approximately 25) compared to the current study, which was based exclusively on MRI-guided transrectal biopsies (approximately 2 probes per lesion) [23]. Nevertheless, regardless of the number of biopsies per patient/core, both studies define a confirmed lesion by at least one positive biopsy. Roethke et al. [23] report their results in patient-based percentages, in contrast to our study which is needle-based. Taking into account the different reporting methods, our results are technically comparable with the needle-based results of Roethke et al., i.e. sensitivity/specificity 54.67%/97.76%.

Another methodological variation that might have influenced the discrepancy from previous work is that Roethke et al. implement a MAI-max cut-off value of 0.6 as criterion for malignant lesion classification [23]. This method, though more objective than visual classification, was not applicable in our study because almost all lesions showed a maximum MAI value higher than 0.6 (Supporting information, S1).

A prerequisite of the high CAD-accuracy is the training of the classifier on a database with similar characteristics to the testing database [13]. An important limitation of this study is the lack of interaction with the classifier of the commercially available tested CAD-software [23]. Despite the classifier having been trained on scanner data with the same (3T) field strength, factors such as the different technical characteristics, coils, static magnetic field inhomogeneities and protocols for the resonance frequency adjustment led to contrast differences that could sufficiently affect the outcome. In a thorough review by Wang et al. [13], numerous studies with databases varying between 15 and 100 patients were compared, not only in terms of performance but also in terms of the analyzed modalities, field strength, ground truth, the method for candidate lesion generation and applied classifier, revealing a broad heterogeneity. Implementation of different receiver coils, such as the use of endorectal coil [33,34], increases the methodological variation.

Moreover, variations in the applied DWI b-values, i.e. b 0–800 s/mm^2^ in a previous study [23] compared to b 0–1000 s/mm^2^ in our study, could affect the ADC computation and inject a significant classification error probability [32,34]. ADC values are widely accepted as the most relevant malignancy feature and rank highly in CAD-classifiers [27–32]. Interestingly, previous reports [37–40] have suggested the superiority of b values of 1000 and 1500 s/mm^2^ compared to either 300, 500 or 2000 s/mm^2^.

The lesion volume should be considered as an independent factor. The current study had, as a single exclusion criterion, technical data incompatibility with Watson Elementary^™^. In ca. 86% of the cases, the sampled lesion´s volume was smaller than 1.0 ml; and in 49%, smaller than 0.5 ml, in keeping with early PCa diagnosis. Watson Elementary^™^ showed promising performance only in lesions larger than 1.0 ml, which might explain differences with previous studies where volume inclusion criteria might have differed. In the studies of Roethke et al. [23,41], lesions smaller than 0.5 ml were not considered clinically significant cancer, in line with ESUR guidelines 2012 [17]. However, lack of methodological definition on volume selection criteria does not allow for a more elaborated comparison. It is remarkable that both the malignity and Gleason grade of smaller, „clinically insignificant”lesions do not differ compared to larger lesions, as shown in Fig 7C. This result supports the existing body of evidence that small cancers can significantly affect a patient´s outcome and encourages biopsy and treatment according to guidelines [42].

Furthermore, lack of access to whole-mount prostate pathology was a limitation of this study with possible influence on the results. The classifier of Watson Elementary^™^ has been regularized to create congruence with malignity grade, a.k.a. Gleason grade [23]. Nonetheless, MAI did not significantly correlate with the pathological outcome, which is the Gleason score in our database. Inter-observer differences in Gleason grading between the training and the testing database could already contribute to this discrepancy, albeit minimally, as previous studies have shown negligible inter-observer variation mostly at the upper and lower limit, namely 4 and 8–10 of the Gleason scale [43,44]. Another possible variability factor may rely on the classifier´s training on whole slide pathology specimens, whereas the grading process in the current study was based on needle biopsies [45,46]. Moreover, the unequal sample distribution with the majority of patients revealing Gleason 6 or 7 (3+3 or 3+4) at the time of diagnosis may bias Pearson´s correlation coefficient negatively [47]. However, such an unequally weighted Gleason distribution is the typical occurrence pattern in population screening and should be taken into account in the training process of a detection method [48,49].

The ROC—analysis of the observer´s performance reveals a reduced accuracy of our study compared with previously published results on PI-RADS [16,41]. Kasel-Seibert et al. In a recent study, compared the performance of PI-RADS version 2 to version 1, showing a high accuracy of AUC of 0.88 and 0.91 for v1 and v2, respectively, in the hands of experienced MRI readers [50]. However, considerable differences in the methodology and database selection should be taken into consideration. Patients with PI-RADS 1 or 2 were not subjected to biopsy and therefore not included in our study. The majority of PI-RADS 3 patients was also not biopsied in our hospital, and were therefore excluded from this study. A limited number of included PI-RADS 2 and 3 lesions derived mostly by downgrading PI-RADS v1 4 lesions in the re-evaluation process after the introduction of PI-RADS v2. On the other hand, Kasel-Seibert et al. [50], as well as other previous studies [26] included PI-RADS 3 lesions in their official selection criteria. Another considerable bias-introducing factor, as the authors also acknowledge, is the patient selection criteria (systematic biopsy was not performed) and the relative low (29%) malignancy rate within the selected population [50]. In the current study, almost all patients were subjected to a (non conclusive or negative) systematic biopsy and the malignancy rate was ca. 45%, thus considerably higher compared to previously published data [50].

Recently, studies that evaluate the role of CAD implementation in improving the radiologist´s performance have been gaining ground on those evaluating stand-alone performance, such as our study [51,52]. Large scale approaches (n = 89 [51] and n = 107 [52]) implemented different methodologies to show that CAD implementation improved radiologists’ sensitivity from 80.9% to 87.6% [51]. Accuracy without and with CAD-combined reading for differentiation between benign and malignant (AUC 0.81 versus 0.88), indolent and aggressive lesions (AUC 0.78 versus 0.88) was improved, respectively [52]. In both original research works, the CAD classifier has been previously established and trained on a comparable database, in contrast to our study where interaction with the classifier was not possible.

This study shows that a carefully designed commercialized CAD software (Watson Elementary^™^) does not perform satisfactorily when tested with a different instrumentation and imaging configuration, despite using almost double the number of patients compared to previous studies [13,23,33–35]. Lack of whole-mount prostate pathology, the low number of PI-RADS 2 and 3 lesions and the challenging character of the database including small lesions, not necessarily encountered as significant in previous studies, should be encountered as possible limiting factors. It is worth mentioning that the scanning parameters applied in our department fulfill the recommendations for diagnosis as defined by the American College of Radiology in PI-RADS^™^ v2. In line with previous observations reviewed by Wand et al. [13], the results of this study support that super-optimistic CAD-performances might be dataset-bound. Altogether, while being in the right framework, the tested software is not satisfactory yet. A necessary requirement of a CAD-software is the ability to apply and generalize to different scanning settings. A broader, optimally multicenter pool of datasets for broader and maybe interactive classifier training should be implemented to improve the general applicability of CAD systems [13].

## Supporting information

S1 File Original data and metadata in.xlsx spreadsheets. (XLSX)
